# Evaluation of the Swedish Web-Version of Quality of Recovery (SwQoR): Secondary Step in the Development of a Mobile Phone App to Measure Postoperative Recovery

**DOI:** 10.2196/resprot.5881

**Published:** 2016-09-28

**Authors:** Karuna Dahlberg, Maria Jaensson, Mats Eriksson, Ulrica Nilsson

**Affiliations:** ^1^School of Health SciencesÖrebro UniversityÖrebroSweden

**Keywords:** mHealth, ambulatory surgical procedures, postoperative period, mobile phones

## Abstract

**Background:**

The majority of all surgeries are performed on an outpatient basis (day surgery). The Recovery Assessment by Phone Points (RAPP) app is an app for the Swedish Web-version of Quality of Recovery (SwQoR), developed to assess and follow-up on postoperative recovery after day surgery.

**Objectives:**

The objectives of this study are (1) to estimate the extent to which the paper and app versions of the SwQoR provide equivalent values; (2) to contribute evidence as to the feasibility and acceptability of a mobile phone Web-based app for measuring postoperative recovery after day surgery and enabling contact with a nurse; and (3) to contribute evidence as to the content validity of the SwQoR.

**Methods:**

Equivalence between the paper and app versions of the SwQoR was measured using a randomized crossover design, in which participants used both the paper and app version. Feasibility and acceptability was evaluated by a questionnaire containing 16 questions regarding the value of the app for follow-up care after day surgery. Content validity evaluation was based on responses by day surgery patients and the staff of the day surgery department.

**Results:**

A total of 69 participants completed the evaluation of equivalence between the paper and app versions of the SwQoR. The intraclass correlation coefficient (ICC) for the SwQoR was .89 (95% CI 0.83-0.93) and .13 to .90 for the items. Of the participants, 63 continued testing the app after discharge and completed the follow-up questionnaire. The median score was 69 (inter-quartile range, IQR 66-73), indicating a positive attitude toward using an app for follow-up after day surgery. A total of 18 patients and 12 staff members participated in the content validity evaluation. The item-level content validity index (I-CVI) for the staff group was in the 0.64 to 1.0 range, with a scale-level content validity index (S-CVI) of 0.88. For the patient group, I-CVI was in the range 0.30 to 0.92 and S-CVI was 0.67. The content validity evaluation of the SwQoR, together with three new items, led to a reduction from 34 to 24 items.

**Conclusions:**

Day surgery patients had positive attitudes toward using the app for follow-up after surgery, and stated a preference for using the app again if they were admitted for a future day surgery procedure. Equivalence between the app and paper version of the SwQoR was found, but at the item level, the ICC was less than .7 for 9 items. In the content validity evaluation of the SwQoR, staff found more items relevant than the patients, and no items found relevant by either staff or patients were excluded when revising the SwQoR.

## Introduction

Day surgery (outpatient surgery) is an expanding and well-established practice in the United States and in many European countries [1-3]. In the United States and United Kingdom, day surgery accounts for 70% to 75% of all elective surgical procedures [2,3]. Similar trends are seen in Sweden where National statistics show that the majority of surgical procedures are performed in day surgery settings (approximately two million per year), with no age restrictions for day surgery treatments [1]. Day surgery is usually defined as surgery performed on a patient who is admitted and discharged from the hospital on the same day, but can also include surgical procedures where the patient is discharged within 24 hours of the surgery [3]. Patients may experience several symptoms after surgery, such as pain, nausea, vomiting, dizziness, fatigue [4], sore throat, back pain, headache, urinary retention, coldness/shivering [5], and postoperative cognitive dysfunction [6]. After discharge from day surgery, patients are expected to take care of their own recovery by themselves or with relatives [7,8]. Many patients feel that the care given while in the hospital is very good, but feel a lack of professional support after discharge. This includes not knowing how to access help and support, and not getting the help that is needed and expected. Since the majority of day-surgical patients are sent home within the same day, it is important to empower them and their relatives to manage the postoperative recovery [8].

The access to reliable and validated instruments to measure and evaluate the quality of postoperative recovery is important in both research and clinical practice. Furthermore, an assessment of recovery can lead to reduced readmissions to the hospital after day surgery [9]. The Swedish version of Quality of Recovery has recently been developed as a Web-based version; the Swedish Web-version of Quality of Recovery (SwQoR), and it has been used to create a Web-based mobile phone app called Recovery Assessment by Phone Points (RAPP) [10]. RAPP assesses postoperative recovery and is used for follow-up after day surgery; however, there have been no studies examining the content validity of the items in the SwQoR or the feasibility of an app in this context. The objectives of this study are (1) to estimate the extent to which the paper and app versions of the SwQoR provide equivalent values; (2) to contribute evidence as to the feasibility and acceptability of a mobile phone Web-based app for measuring postoperative recovery after day surgery and enabling contact with a nurse; and (3) to contribute evidence as to the content validity of the SwQoR.

## Methods

The first phase of this study was cross-sectional to estimate equivalency of the two versions of the SwQoR; for this estimate the order of the versions were randomized. The second phase used a prospective design. The study was approved by the regional ethical review board in Uppsala, Sweden (2014/456).

### Recruitment

The study was carried out from January to May, 2015. A total of 70 participants were recruited consecutively in two day surgery settings in Sweden. The inclusion criteria were that patients must be adults over 17 years of age, be admitted for day surgery, be able to understand the Swedish language both in speech and in writing, and undergo general anesthesia. The exclusion criterion was that the patient does not having access to a mobile phone with Internet access and a Web browser (smartphone).

For evaluating the content validity of the SwQoR, 18 patients participating in the study and 12 staff members were recruited. The staff group worked in the day surgery departments participating in this study and included 4 anesthesiologists, 4 surgeons, and 4 nurses.

### Mobile Phone App

**Figure 1:**
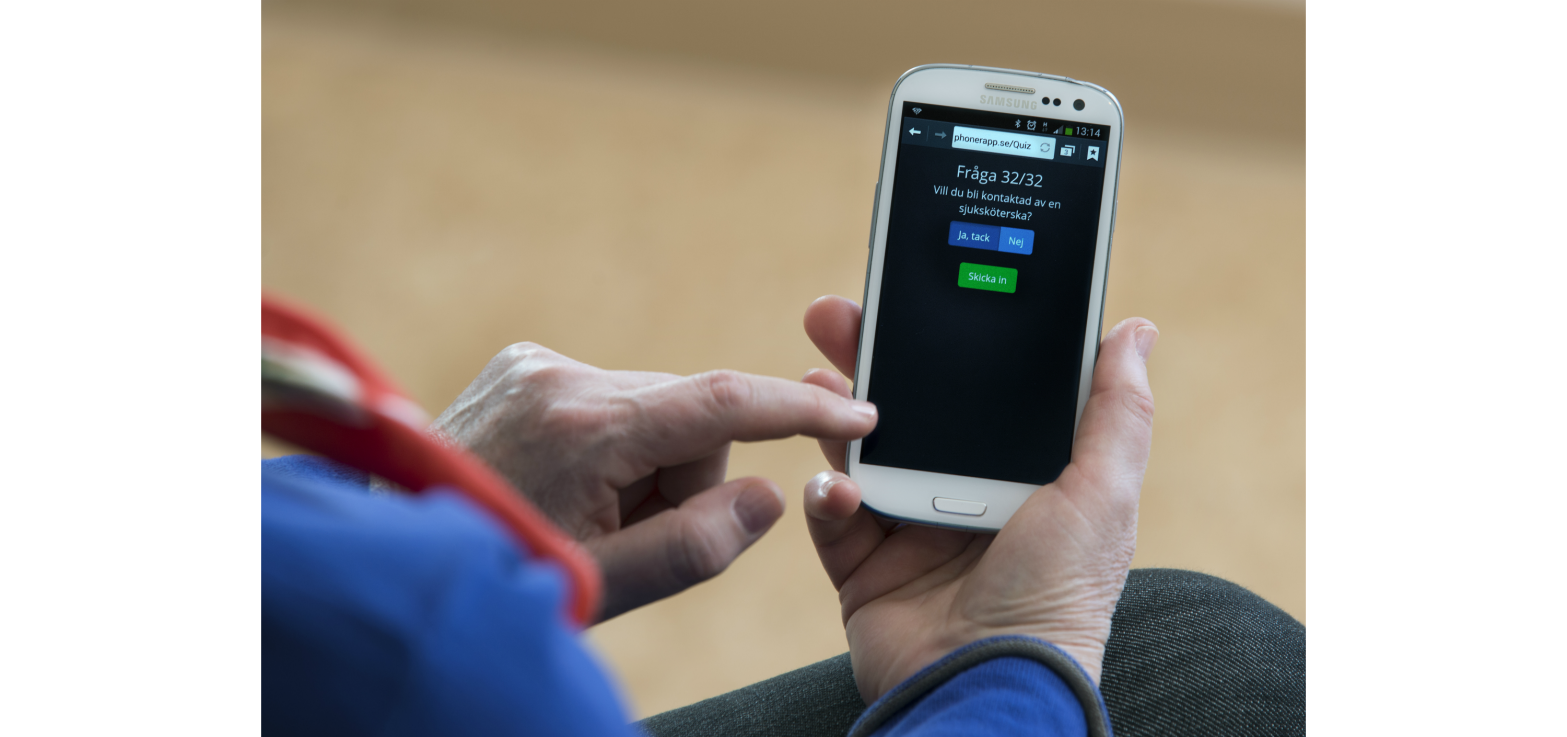
The final question in the app ”Do you want to be contacted by a nurse?”. To be answered with ”Yes” or ”No”.

RAPP is a Web-based app that is suitable for all mobile phone models. The participants’ own mobile phones were used for this study, following the principle of bring your own device (BYOD) [11]. The mobile phone app contained the 31 items in SwQoR, which are answered using an 11-point numeric visual analog scale (VAS). On this scale, 0 represents “none of the time” and 10 “all of the time”. In the app, only one item at a time is visible on the screen, and after the item is answered, the next item automatically appears. After an item is answered, it is not possible to go back to the previous item to review or change the answer. Every day that the patient answered the SwQoR items in the app, the final question was always, “Do you want to be contacted by a nurse?” The patient has to answer “Yes” or “No” to this question [10] (Figure 1).

### Procedure

When making the appointment for their operation, preoperative patients were provided with written information about the study and were requested to bring their mobile phone to the day surgery department on the day of surgery. At admission, the participants were verbally informed about the aim of the study, and those who agreed to participate provided informed consent. Both preoperatively and prior to discharge from the hospital, the participants were thoroughly trained and informed about the app’s functionalities and navigation, and they learned how to document their postoperative recovery. Inclusion, information, and follow-up were conducted by one member of the research team (KD). If the question “Do you want to be contacted by a nurse?” was ever answered with “Yes” by a patient, an immediate email was sent with the participant’s study code to one member of the research group who had access to the code set. This research team member then contacted a nurse at the day surgery department where the surgery was performed, and the nurse contacted the participant. In one of the day surgery settings, all the requested contact calls were conducted by one specific nurse, and in the other setting, the nurse in charge of all incoming phone calls that day made contact with the patient.

### Equivalence Between the App and Paper Version of the SwQoR

To measure the equivalence between the app and paper versions of the SwQoR, participants were randomized into one of the following two conditions for answering its 31 items: (1) a paper questionnaire followed by the app questionnaire, or (2) the app questionnaire followed by the paper questionnaire. In both cases, 30 minutes elapsed between the app and paper measurements for both groups. This interval was guided by an earlier study conducted by Gower et al [12], which compared answers from the Quality of Recovery (QoR) questionnaire between a self-administered and a staff-administered survey. Here, the randomization was accomplished using sealed envelopes in a random order. The randomization was not blinded; both the participants and the researcher had knowledge of the condition assignment for each participant. From 2 to 5 hours after surgery, when the patient was ready for discharge, the app was installed on the participants’ own mobile phone. Then the participants responded to the SwQoR questionnaire in the order according to the randomization.

After completing the first version of SwQoR, the participants were not able to see their previous answers when they responded in the second round.

### Evaluating Feasibility and Acceptability

All included participants were asked to use the RAPP (answer the 31 items in the SwQoR, as well as the final question “Do you want to be contacted by a nurse?”) each day for 7 days after discharge. One member of the research team (KD) was always available during the study period (both by phone and by email) if the participants had any problems using the app. On the 7th day, a follow-up phone call was made by a member of the research team (KD), who used a questionnaire to ask the participants for feedback on using a mobile phone app to assess their postoperative recovery. The follow-up questionnaire was designed for this study and guided by a similar questionnaire used by Ainsworth et al [13] to compare a mobile phone app with text messaging to assess mental illness. The follow-up questionnaire included 16 questions, of which 11 statements were rated from 1 (“Strongly agree”) to 7 (“Strongly disagree”). Examples of the statements were: “Answering the questions took a lot of time”, “I would like to avoid answering the questions”, and “This type of systematic follow-up helped me and would help other patients in the same situation” *.* If the participants requested contact by a nurse via the app, the interviewer asked about the reason for the contact. All participants were also asked about how they experienced the opportunity to get in contact with a nurse via the app. Overall comments regarding the app were obtained, as well as the participants’ opinions about how many days it would be useful to answer the questions in the app during the postoperative period. Finally, the participants were asked if there were any questions that were not asked in the SwQoR that they thought should be included.

Questions that were suggested as missing yet relevant for postoperative recovery were included in the content validity review described below.

### Content Validity of the SwQoR

To assess the content validity of the SwQoR used after a day surgery, staff and patients evaluated the SwQoR together with the items suggested by the participants as missing in the follow-up. The staff members and patients rated the items regarding intelligibility and relevance on a 4-point scale with 1 representing *not relevant*, 2 *somewhat relevant*, 3 *quite relevant*, and 4 *highly relevant*. The content validity assessment was conducted with pen and paper. The patients performed the content validity assessment 1 to 2 weeks postoperatively (ie, after the testing of the app was completed).

### Confidentiality and Security

Each participant was assigned a study code and no personal data, such as social security number, name, age, gender or telephone number were stored in the app. Only one member of the research team had access to the code set and could identify who was answering the app. The paper questionnaires were also coded. The codes were stored separately from the questionnaires. Data transmission between the mobile phone and the server used for the test occurred via the mobile network General Packet Radio Service (GPRS), and the data were stored in a secure server that required a login and password to access the answers from the app.

### Statistical Analysis

The equivalence testing between the paper and app versions was analyzed using the intraclass correlation coefficient (ICC) (one-way, single measures). An ICC value of .7 or above was considered acceptable [14]. Item-by-item differences between the paper and app versions were compared using Wilcoxon´s signed rank test, and the null hypothesis was rejected if the two-tailed *P* value was less than .01. Internal consistency was estimated by Cronbach’s alpha, where .90 was considered a minimum value for clinical applications [15]. Results from the follow-up questionnaire were presented as descriptive statistics and were expressed as median, inter-quartile range (IQR), and min-max. The content validity of the SwQoR was presented as frequencies and the content validity index. The item-level content validity index (I-CVI) (ie, the number of participants who rated the item either 3 or 4), was calculated. It has been suggested that I-CVI should be at least 0.78 (with more than 6 participants) to indicate good content validity, and the scale-level content validity index (S-CVI) (average of all I-CVI) should be 0.9 or higher [16]. SPSS statistics version 22 (SPSS Inc., Chicago, IL, USA) for Windows was used for the statistical analyses.

### Power

To our knowledge, no previous studies have compared paper and digital/electronic postoperative questionnaires. Thus, the number of participants for this study was guided by two earlier studies in other contexts that compared paper and electronic questionnaires. Salaffi et al [17] included 55 adult participants with axial spondyloarthritis and compared their answers on a paper-based patient reported outcome (PRO) questionnaire to answers using a touch screen tablet. Furthermore, Bushnell and colleagues [18] performed a similar comparison with 72 adult participants suffering from irritable bowel syndrome. In both studies, equivalence was shown between the two measurement formats; based on these results, the present study included 70 participants.

## Results

**Figure 2:**
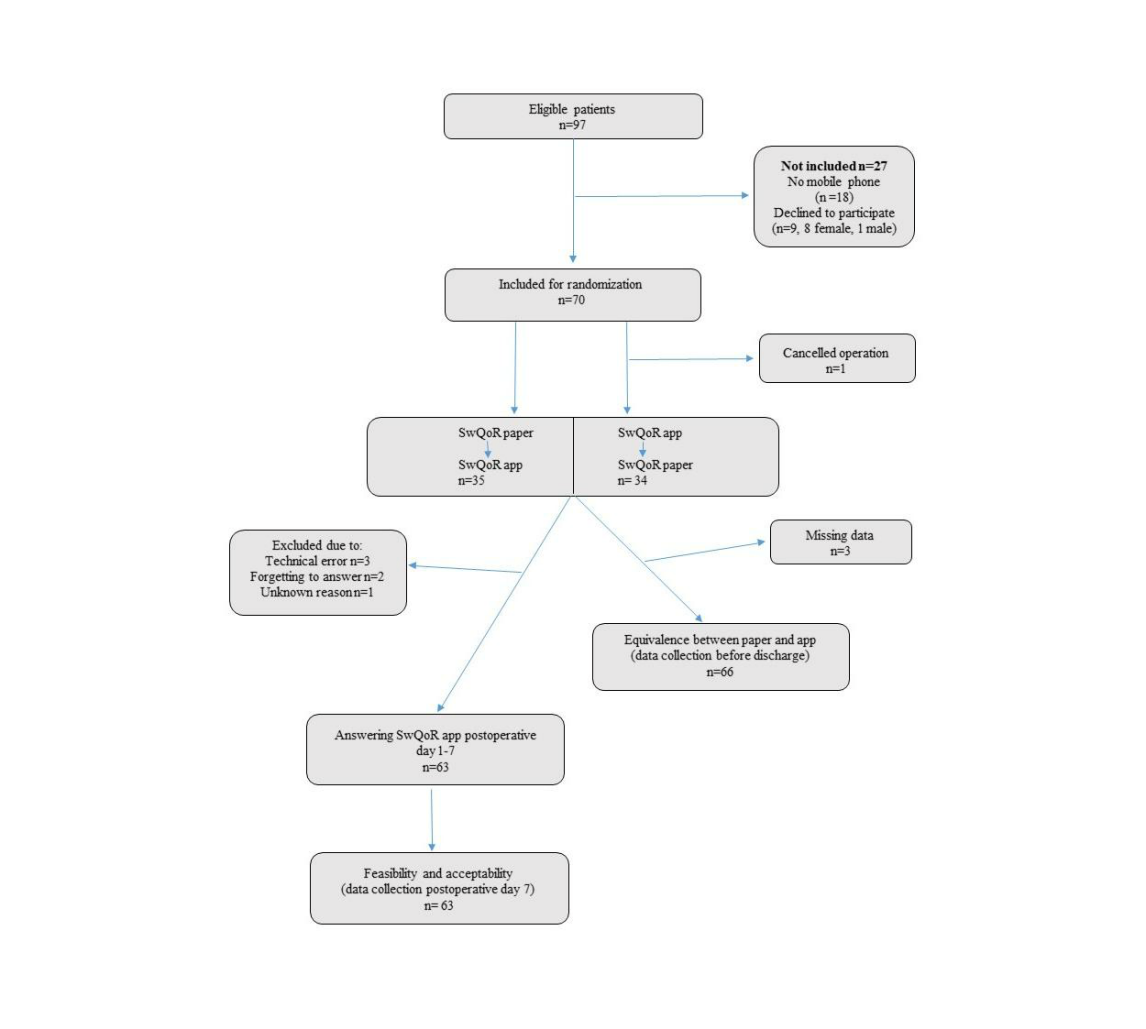
Flowchart describing the recruitment of participants and data collection.

Surgery was canceled for one of the 70 included participants. The characteristics of the remaining 69 patients are presented in Table 1. All participants started to answer the SwQoR according to the order determined by the randomization (ie, app or paper version first). Two participants could not submit the app answers due to technical problems (inability to connect to the network or log in). One participant misunderstood how to fill in the app version of the SwQoR (reporting opposite answers than in the paper version, not understanding that the scale was intact when items shifted from positive to negative) and was excluded from the equivalence testing between the app and paper version of the SwQoR. The technical issues and misunderstanding were solved and all three participants were then able to use the app from postoperative day one and thus contribute to the feasibility and acceptability testing of the app. In addition, 6 participants did not complete the feasibility and acceptability evaluation; 3 due to technical error, 2 for forgetting to answer, and 1 for an unknown reason, giving a total of 63 participants (Figure 2).

**Table 1:** Patient characteristics (N=69).

Characteristics		n (%)
Sex		
	Men	41 (59)
	Women	28 (41)
Age, mean (SD)		50 (15)
Surgery type		
	General	33 (48)
	Orthopedic	26 (38)
	Gynecology	4 (6)
	Hand	3 (4)
	Ear, nose, throat	3 (4)

### Equivalence Between the App and Paper Versions of SwQoR

The agreement between the app and paper versions is presented in Table 2. The ICC for the total scale was .89 (95% CI 0.83-0.93) and it was in the range .13 to .90 for the items. The differences between the app and paper versions of the items in the SwQoR were not statistically significant except for three items (Table 2). Cronbach´s alpha was .91 for the app version and .91 for the paper version.

**Table 2:** Agreement between the app and paper versions of the SwQoR (N=66).

Category	Paper median^a^ (IQR)	App median (IQR)	*P* value^b^	ICC (95% CI)
Able to breathe easy	10 (9.75-10)	10 (9.75-10)	.25	.81 (0.71-0.88)
Sleeping well	10 (8-10)	10 (8-10)	.28	.89 (0.82-0.93)
Being able to enjoy food	10 (8-10)	10 (8-10)	.12	.80 (0.69-0.87)
Feeling rested	9 (7-10)	8 (5-10)	.008	.80 (0.67-0.87)
Having a general feeling of well-being	9 (8-10)	9 (6-10)	.03	.73 (0.59-0.83)
Feeling in control	9 (8-10)	9 (7-10)	.75	.82 (0.72-0.88)
Feeling relaxed	9 (8-10)	9 (7-10)	.14	.77 (0.65-0.85)
Speaking normally	10 (9-10)	10 (8-10)	.013	.71 (0.57-0.81)
Able to look after personal hygiene	10 (9-10)	10 (8-10)	.63	.68 (0.53-0.80)
Able to write as usual	10 (10-10)	10 (9-10)	.04	.86 (0.78-0.91)
Able to return to work or usual duties about the home	5 (1-10)	5 (2-9)	.82	.90 (0.84-0.94)
Nausea and/or vomiting	0 (0-3)	0 (0-2.75)	.06	.89 (0.82-0.93)
Feeling restless	0 (0-1)	0.5 (0-4)	.001	.49 (0.28-0.66)
Shivering or twitching	0 (0-0)	0 (0-1)	.003	.36 (0.12-0.55)
Feeling too cold	0 (0-3)	1 (0-3)	.20	.76 (0.64-0.85)
Dizziness	1 (0-4.75)	1.5 (0-5)	.23	.61 (0.43-0.75)
Pain in the surgical wound	2.5 (0.25-6.75)	3 (0-7)	.27	.72 (0.57-0.82)
Anxiety	0 (0-2)	0 (0-2)	.87	.66 (0.50-0.78)
Depressed	0 (0-1)	0 (0-1)	.16	.80 (0.69-0.87)
Feeling lonely	0 (0-0)	0 (0-1)	.03	.68 (0.53-0.79)
Difficulties getting to sleep	0 (0-1)	0 (0-1.25)	.82	.64 (0.48-0.77)
Nightmares	0 (0-0)	0 (0-0)	.02	.87 (0.80-0.92)
Headache	0 (0-2)	0 (0-2)	.39	.81 (0.71-0.88)
Muscle pain	0 (0-2)	0 (0-2)	.67	.74 (0.61-0.83)
Back pain	0 (0-0)	0 (0-1)	.07	.83 (0.74-0.89)
Sore throat	0 (0-1)	0 (0-2)	.02	.79 (0.68-0.87)
Sore mouth	0 (0-0)	0 (0-0.25)	.05	.78 (0.66-0.86)
Difficulties concentrating	0 (0-2)	0 (0-2)	.22	.78 (0.66-0.86)
Trouble urinating	0 (0-0)	0 (0-0)	.37	.73 (0.59-0.83)
Diarrhea	0 (0-0)	0 (0-0)	.07	.30 (0.06-0.51)
Feeling constipated	0 (0-0)	0 (0-0)	.03	0.13 (-0.12-0.36)

^a^0= none of the time, 10=all of the time.

^b^Wilcoxon signed ranks test.

### Feasibility and Acceptability

The RAPP was answered over a mean of 5 days (min 1, max 7). When asked about reasons for not answering the RAPP all 7 days, 8 participants reported not remembering to answer, 5 reported technical issues such as the app logging out or problems with the network, and 2 were re-admitted to the hospital. Those participants who forgot to answer declared that they wanted a daily reminder.

Results from the follow-up questionnaire showed that the participants had a positive attitude toward using the app, felt comfortable using the technology, and took a reasonable amount of time to answer the items in the app (Table 3). On average, the participants considered that 9 days (min 3, max 60) would be acceptable for measuring postoperative recovery after day surgery via an app. They also expressed that the items should be either all positive or all negative to make it easier to answer on the numeric VAS. This would allow the good/bad rating to be on the same side of the scale for all items, thus decreasing the risk of answering falsely.

**Table 3:** Results from the follow-up questionnaire, questions 1 to 11 (N=63).

Question	Median^a^	IQR	Min, max
I felt familiar with using this type of technology	1	1-1	1, 3
I would like to use this type of postoperative follow-up again if undergoing surgery	1	1-1	1, 4
I think other people would find the software tool easy to use	2	1-3	1, 5
This type of systematic follow-up helped me and would help other patients in the same situation	1	1-2	1, 4
Answering the questions made me feel better	5	3-7	1, 7
It was difficult to answer the questions	7	7-7	2, 7
I would like to avoid answering the questions	7	7-7	2, 7
Answering the questions took a lot of time	7	7-7	4, 7
It was difficult to keep track of what the questions were asking	7	6-7	4, 7
It was inconvenient to answer the questions using my smartphone	7	7-7	2, 7
Answering the questions made me feel worse	7	7-7	1, 7
Total score^b^ (positive items reversed)	69	66-73	45, 77

^a^1= strongly agree, 7=strongly disagree.

^b^Minimum possible score 11, maximum possible score 77.

The request to be contacted by a nurse via the app was used 15 times (3.4%, 15/441) in relation to the total number of chances to request contact (441 instances with 63 participants using the app for 7 days each). The reasons for the contact were the following: (1) questions concerning the surgical wound regarding the dressing, stitches, swelling, etc (44%, 7/16), (2) pain and/or pain management (19%, 3/16), (3) general information (13%, 2/16), (4) constipation (13%, 2/16), (5) request for a medical certificate (6%, 1/16), and (6) nausea (6%, 1/16). The opportunity to get in contact with a nurse via the app provided a sense of security and was appreciated by all except one of the participants, who wanted to use the telephone for initiating contact instead of the app. Participants (25%, 16/63) also expressed that it is typically difficult to contact a caregiver and that this opportunity provided a simple solution for that problem. Three additional items were suggested by the participants: fever, reddened surgical wound, and swollen surgical wound.

### Content Validity of the SwQoR

In total, 34 items were included when evaluating content validity (ie, the original 31 items and 3 additional items). Five surveys from the patients were incomplete, 13 (72%, 13/18) were included in this analysis. Results of the content validity are presented in Multimedia Appendix 1 with I-CVI and S-CVI. The I-CVI values for the staff group were in the range 0.64 to 1.0 (S-CVI 0.88), and the I-CVI values for the patient group were in the range 0.30 to 0.92 (S-CVI 0.67). The staff group rated all items higher than the patient group ratings.

### Revising Items in the SwQoR

An I-CVI rating less than 0.78 by both patient and staff led to the removal of the following 7 items in the SwQoR: (1) able to enjoy food, (2) able to write, (3) feeling restless, (4) shaking or twitching, (5) feeling too cold, (6) feeling alone, and (7) backache. When calculating the S-CVI after removing these 7 items, the S-CVI was 0.94 for staff and 0.72 for patients. Four related items that were considered by patients to be very similar (had a good sleep, feel rested, had difficulty falling asleep, had bad dreams *)* were merged into one item: sleeping difficulties. Thus, guided by the results of the CVI and the feedback from the patients, the 34 items were reduced to 24 (Table 4).

**Table 4:** Revision of items in SwQoR.

Revision	SwQoR 31	SwQoR 24
Merged into one item	Sleeping well	Had sleeping difficulties
	Nightmares	
	Difficulties getting to sleep	
	Feeling rested	
Not changed	Able to breathe easy	Able to breathe easy
	Having a general feeling of well-being	Having a general feeling of well-being
	Feeling in control	Feeling in control
	Feeling relaxed	Feeling relaxed
	Speaking normally	Speaking normally
	Able to look after personal hygiene	Able to look after personal hygiene
	Able to return to work or usual duties about the home	Able to return to work or usual duties about the home
	Nausea and/or vomiting	Nausea and/or vomiting
	Dizziness	Dizziness
	Pain in the surgical wound	Pain in the surgical wound
	Anxiety	Anxiety
	Depressed	Depressed
	Headache	Headache
	Muscle pain	Muscle pain
	Sore throat	Sore throat
	Sore mouth	Sore mouth
	Difficulties concentrating	Difficulties concentrating
	Trouble urinating	Trouble urinating
	Feeling constipated	Feeling constipated
	Diarrhea	Diarrhea
Included after content validity assessment	N/A	Reddened surgical wound
		Fever
		Swollen surgical wound
Excluded after content validity assessment	Feeling restless	N/A
	Shivering or twitching	
	Feeling too cold	
	Being able to enjoy food	
	Able to write as usual	
	Back pain	
	Feeling lonely	

## Discussion

### Principal Findings

The present study shows agreement between the paper and app versions of the SwQoR, but on an item level in the SwQoR, the ICC was less than 0.7 for 9 items. The participants were very positive toward using the app for a follow-up survey after undergoing day surgery, did not find it to take too long to fill in, and were willing to use this follow-up method if admitted for a future day surgery. The content validity showed that more items were found to be relevant by the staff group compared to the patient group.

When measuring equivalence between the paper and app version of the SwQoR, we used parametric statistics even though the SwQoR collects ordinal level data. This allows results from the study to be compared with results from previous studies on the QoR instrument [12,19-21]. Electronically assessed PROs have been shown to be at least equivalent to those from a paper assessment, but it was suggested that for every PRO converted from paper to digital format, the equivalence should be measured [22]. In this study, the ICC between the paper and app version of the SwQoR scale was excellent (ICC .89). This is similar to earlier results that reported test-retest values for QoR with ICC values of .92 [21] and .99 [20], a Spearman’s correlation coefficient of .89 [23], and ICC .86 when measuring equivalence in patient- versus investigator-administered QoR 40 [12]. At the item level, however, the ICC was less than .7 for 9 items, and the difference was significant for 3 items. This is the first time that the ICC for each item is being presented regarding the QoR instrument, so there is no guidance from previous studies. One reason for the low ICC for some items could be that the app permits someone to accidentally answer with the default value before getting the chance to select a different value. Selecting a value is accomplished by moving a dot on the numeric VAS, and the dot is stationed in the center (at a value of 5) for every new item. However, the app allows a user to push the answer button without moving the dot, and this could have led to falsely reported values. Further, a low ICC was also noted in the items following after the items shifted from positive to negative (ie, a positive item being “Able to look after personal hygiene”, and negative item being “Feeling restless”). This change was visually clearer in the paper version of SwQoR, since in the app only one question at a time is visible on the screen. The participants also expressed that it was hard to follow when the direction of the question shifted from positive to negative (ie, a positive answer is sometimes indicated on the right side of the scale and sometimes on the left side). Furthermore, it was not possible to go back and change prior answers without starting from the beginning. Some patients, after answering wrongly, would start again from the beginning to report their answers, but it was expressed that this was cumbersome and time-consuming. Three of the items with ICC less than .7 were excluded after the content validity evaluation. This indicates that changes in the layout of the items in the app may result in greater agreement on the item level than that achieved in this study.

In our study, both the app and paper version of the SwQoR showed excellent internal consistency (Cronbach’s alpha .91 and .91, respectively), which is a similar result to previous studies regarding the QoR with Cronbach’s alpha values of .93 [21], .95 [24], .93 [25], and .85 [20].

To our knowledge, there has been only one previous study that tested an app for follow-up after day surgery. Semple at al [26] followed orthopedic and breast reconstruction patients admitted for day surgery who participated in postoperative follow-up via an app. Of the participants, 87% reported that the overall experience using the app was excellent and they were willing to use the same technology if undergoing surgery again [26]. This is similar to the results in our study, where the participants found the app easy to use, thought that the systematic follow-up was helpful in the postoperative period, and wanted to use the app for follow-up after future day surgeries.

Similar results were also reported in a study by Stomberg et al [27] assessing pain for one week postoperatively via a paper-based questionnaire or a mobile phone app. Overall, the participants were willing to use the mobile phone assessment again. Further, the participants in our study wanted to use the app on average 9 days postoperatively (ie, longer than the duration requested in the study). Electronic questionnaires are described to be a user-friendly method [28] and preferred by many patients. They also result in less missing data compared to data collected by conventional pen-and-paper questionnaires [27]. In our study, the participants expressed that the ability to get in contact with a nurse via the app was one of its most valuable features. Participants expressed that, otherwise, it was difficult to get in contact with a caregiver due to not knowing who to call, what number to use, or what time to call. The contact function in the app was considered an easy solution to this problem. Further, participants expressed that the opportunity to get in contact with a nurse made them feel secure. This was also described in a study by Berg et al [29], in which patients felt more secure when there was an easy way of getting in contact with the caregiver by telephone. The results in the present study support the hypothesis and aim that the RAPP would contribute to both “a feeling of being cared for” and “being easy to understand for patients in the health care system”, which are described by Jaensson et al [10].

The principle of BYOD was used in this study, which resulted in excluding 18 otherwise eligible patients. This could of course have affected the results regarding feasibility and acceptability, since it would be natural that a person with access to a mobile phone would have a more positive attitude toward using mobile apps. However, the number of mobile phone users is increasing and will most likely continue to increase in coming years. Further, a BYOD-approach eliminates the cost of providing tablets or mobile phones (smartphones) in the health care system and in the study [11]. Other advantages are that patients are most familiar with their own mobile phones and will be more likely to have their own devices available most of the time [30].

In the content validity evaluation in this study, the analyses for staff and patients were conducted separately as these two groups utilize the SwQoR assessment from different points of view. Staff members in the day surgery department assess postoperative recovery to follow-up on, evaluate, and improve anesthetic and postoperative care. Patients personally experience the postoperative recovery and thus use the SwQoR to report on that recovery. In the evaluation of content validity, staff rated the items higher than the patients. Only for 9 items was I-CVI greater than 0.78 in the patient group, whereas I-CVI was greater than 0.78 for 27 items in the staff group. The rank order of the items was similar in both the patient and staff groups, as the patients just tended to consider items to be less relevant than the staff in this study. In contrast to our results, Myles at al [31] found that the staff tended to rate the relevance of the items lower than the patients and their relatives. However, in the Myles study, the items were rated by inpatients and their relatives. In 50% of the cases, the ratings were made preoperatively [31]. In our study, patients performed the content validity assessment postoperatively and after testing the app for 7 days. This would probably lead to items not being considered relevant in the content validity testing if patients did not experience the symptoms as burdensome in their postoperative period. Macario et al [32,33] described both differences and similarities between patients and anesthesiologists when rating most undesirable anesthesia outcomes (from the patient’s point of view). The results also showed that anesthesiologists considered most of the complications due to anesthesia important to avoid [33], which is similar to the findings from our study, as the staff considered most conditions important enough for a follow-up.

When revising the SwQoR, the decision was made to retain any item with I-CVI greater than 0.78 in either the patient or the staff group, since that item was considered relevant for the perspectives and contexts of each group. Revision of the QoR was previously described by Stark et al [20] in order to improve its clinical acceptability and feasibility and to make it more useful in clinical practice and research. No content validity assessment was reported by Stark et al [20]; their revision was guided by literature studies and consultation with experienced staff, resulting in the 15 item QoR15.

The items in the QoR have been previously summarized and reported across the five dimensions *emotional state*, *physical comfort*, *psychological support*, *physical independence*, and *pain*; these reports indicated the quality of recovery in each dimension [12,19-21,24,25]. However, in this study and its context, the focus was on items, not dimensions. We believe that, when day surgery departments follow-up with their patients, the interest is in each specific item when evaluating and improving anesthetic and postoperative care. For example, when evaluating intravenous versus inhalation anesthesia and the postoperative differences in nausea and vomiting, the follow-up and evaluation would examine the values reported in the item “Nausea and/or vomiting”, not the quality of recovery in the dimension *physical comfort*.

### Limitations

This study was conducted in two day surgery departments in Sweden including participants familiar with using mobile phones and participants who spoke and could read the Swedish language. Only day surgery patients who underwent general anesthesia were included. Further studies including all types of anesthesia and surgeries should be conducted, as well as studies including non-Swedish speaking participants answering in their own language. There was also a technical limitation including no opportunity for the participants to change their prior answers in the app and this might have affected the reported answers.

### Suggestion for Further Development

On the basis of our results, we recommend some changes to be implemented in the next version and further development of the RAPP app (Textbox 1).

**Recommended changes in the next version of the RAPP app**

A value on the numeric VAS should be chosen before the respondent can continue to the next question.

Incorporate the ability to go back and check or change prior answers.

Reformulate all positive (n=8) items into negative items.

Include a daily reminder to fill in the app, which is only possible in a native app.

Develop the web-based app as a native app.

Testing of the new items that was included after the content validity.

### Conclusions

Day surgery patients had positive attitudes toward using this app for follow-up after surgery and wanted to use the app again if admitted for future day surgeries. The ability to get in contact with a nurse via the app was very much appreciated and made the participants feel secure. Equivalence between the app and paper versions of the SwQoR showed agreement (ICC .89), but at the item level, the ICC was less than .7 for 9 items. This study shows the importance of evaluating an instrument converted from paper to electronic assessment formats and the need to evaluate the specific app for this assessment. In the content validity evaluation of the SwQoR, staff found more items relevant than the patients. The content validity evaluation of the SwQoR together with 3 new items led to a reduction from 34 to 24 items in the SwQoR.

## Multimedia Appendix 1

Content validity SwQoR.

## Abbreviations

BYOD: bring your own device
ICC: intraclass correlation coefficient
I-CVI: item-level content validity index
PRO: patient reported outcome
QoR: Quality of Recovery instrument
RAPP: Recovery Assessment by Phone Points
S-CVI: scale-level content validity index
SwQoR: Swedish Web-version of Quality of Recovery
VAS: visual analogue scale
