# Supplementary material for: Evaluation of the Swedish Web-Version of Quality of Recovery (SwQoR): Secondary Step in the Development of a Mobile Phone App to Measure Postoperative Recovery
Source: JMIR Res Protoc. 2016 Sep 28;5(3):e192. doi: 10.2196/resprot.5881 (PMC5051790; doi:10.2196/resprot.5881)
Supplement: Supplementary file 1 [file resprot_v5i3e192_app1.pdf]

Content		4=Highly relevant, n (%)	3=Quite relevant, n (%)	2=Somewhat relevant, n (%)	1=Not relevant, n (%)	I-CVI
Pain in the surgical wound						
	Staff	11 (100)	0	0	0	1.0
	Patient	12 (92)	0	1 (8)	0	0.92
Reddened surgical wound[a]						
	Staff	11 (92)	0	1 (8)	0	0.92
	Patient	10 (77)	2 (15)	1 (8)	0	0.92
Fever[a]						
	Staff	12 (100)	0	0	0	1.0
	Patient	9 (69)	2 (15)	1 (8)	1 (8)	0.84
Nausea and/or vomiting						
	Staff	9 (100)	0	0	0	1.0
	Patient	9 (69)	2 (15)	0	2 (15)	0.84
Trouble urinating						
	Staff	11 (92)	1 (8)	0	0	1.0
	Patient	9 (69)	1(8)	1 (8)	2 (15)	0.77
Able to breathe easy						
	Staff	12(100)	0	0	0	1.0
	Patient	8 (62)	2 (15)	2 (15)	1 (8)	0.77
Swollen surgical wound[a]						
	Staff	8 (67)	2 (17)	2 (17)	0	0.83
	Patient	11 (85)	1 (8)	0	1 (8)	0.92
Dizziness						
	Staff	8 (73)	3 (27)	0	0	1.0
	Patient	8 (62)	2 (15)	2 (15)	1 (8)	0.77
Feeling constipated						
	Staff	8 (67)	4 (33)	0	0	1.0
	Patient	7 (54)	4 (31)	0	2 (15)	0.85
Anxiety						
	Staff	8 (73)	3 (27)	0	0	1.0
	Patient	7 (54)	3 (23)	2 (15)	1 (8)	0.77
Able to return to work or usual duties about the home						
	Staff	9 (82)	2(18)	0	0	1.0
	Patient	7 (54)	2(15)	3 (23)	1 (8)	0.69
Able to look after personal hygiene						

	Staff	6 (50)	6 (50)	0	0	1.0
	Patient	5 (38)	6 (46)	1 (8)	1(8)	0.84
Diarrhea						
	Staff	7 (58)	4 (33)	1 (8)	0	0.92
	Patient	7 (54)	3 (23)	0	3 (23)	0.77
Feeling in control						
	Staff	7 (58)	4 (33)	1 (8)	0	0.92
	Patient	5 (38)	5 (38)	3 (23)	0	0.76
Sleeping well						
	Staff	7 (58)	3 (25)	2 (17)	0	0.83
	Patient	6 (46)	5 (38)	2 (15)	0	0.84
Difficulties getting to sleep						
	Staff	5 (45)	5 (45)	1 (9)	0	0.91
	Patient	6 (46)	5 (38)	0	2 (15)	0.85
Muscle pain						
	Staff	6 (50)	5 (42)	1 (8)	0	0.92
	Patient	5 (38)	5 (38)	1 (8)	2 (15)	0.76
Sore throat						
	Staff	9 (75)	3 (25)	0	0	1.0
	Patient	4 (31)	3 (23)	3 (23)	3 (23)	0.54
Headache						
	Staff	9 (75)	2 (17)	1 (8)	0	0.92
	Patient	5 (38)	3 (23)	2 (15)	3 (23)	0.61
Having a general feeling of well-being						
	Staff	7 (58)	4 (33)	0	1 (8)	0.92
	Patient	2 (15)	8 (62)	3 (23)	0	0.77
Nightmares						
	Staff	5 (45)	6 (55)	0	0	1.0
	Patient	3 (23)	5 (38)	0	5 (38)	0.62
Feeling relaxed						
	Staff	3 (25)	7 (58)	2 (17)	0	0.83
	Patient	3 (23)	5 (38)	4 (31)	1 (8)	0.61
Feeling rested						
	Staff	2 (17)	9 (75)	1 (8)	0	0.92
	Patient	3 (23)	4 (31)	5 (38)	1 (8)	0.54
Depressed						
	Staff	3(27)	7 (64)	1(9)	0	0.91
	Patient	4 (31)	3 (23)	2(15)	4 (31)	0.54
Sore mouth						
	Staff	7 (58)	4 (33)	0	1 (8)	0.92
	Patient	2 (15)	3 (23)	4 (31)	4 (31)	0.38
Difficulties concentrating						
	Staff	6 (50)	5 (42)	1 (8)	0	0.92

	Patient	2 (15)	2 (15)	6 (46)	3 (23)	0.30
Speaking normally						
	Staff	4 (33)	6 (50)	0	2 (17)	0.83
	Patient	4 (31)	3 (23)	1 (8)	5 (38)	0.54
Feeling too cold						
	Staff	2 (17)	6 (55)	2 (18)	1 (9)	0.72
	Patient	6 (46)	2 (15)	3 (23)	2 (15)	0.61
Being able to enjoy food						
	Staff	2 (17)	6 (50)	4 (33)	0	0.67
	Patient	4 (31)	4 (31)	5 (38)	0	0.62
Shivering or twitching						
	Staff	2 (17)	5 (42)	3 (25)	1 (9)	0.59
	Patient	7 (54)	1 (8)	2 (15)	3 (23)	0.62
Back pain						
	Staff	4 (33)	4 (33)	4 (33)	0	0.66
	Patient	2 (15)	5 (38)	4 (31)	2 (15)	0.53
Feeling lonely						
	Staff	3 (27)	5 (46)	2 (18)	1 (9)	0.73
	Patient	2 (15)	3 (23)	3 (23)	5 (38)	0.38
Feeling restless						
	Staff	1 (9)	6 (55)	3 (25)	1 (9)	0.64
	Patient	3 (23)	2 (15)	6 (46)	2 (15)	0.38
Able to write as usual						
	Staff	2 (18)	5 (46)	3 (27)	1 (9)	0.64
	Patient	2 (15)	2 (15)	3 (23)	6 (46)	0.30

[a]New items that emerged from the follow-up questionnaire as questions missing in the SwQoR.
